# Progress toward the Definition of X-ray Computed Tomography Accuracy in the Characterization of Polymer-Based Lattice Structures

**DOI:** 10.3390/polym16101419

**Published:** 2024-05-16

**Authors:** Daniel Gallardo, Lucía-Candela Díaz, José Antonio Albajez, José Antonio Yagüe-Fabra

**Affiliations:** I3A, Universidad de Zaragoza, María de Luna 3, 50018 Zaragoza, Spain; lcdiaz@unizar.es (L.-C.D.); jalbajez@unizar.es (J.A.A.); jyague@unizar.es (J.A.Y.-F.)

**Keywords:** X-ray computed tomography, lattice structures, additive manufacturing

## Abstract

Lattice structures have become an innovative solution for the improvement of part design, as they are able to substitute solid regions, maintain mechanical capabilities, and reduce material usage; however, dimensional quality control of these geometries is challenging. X-ray computed tomography (XCT) is the most suitable non-destructive metrological technique as it is capable of characterizing internal features and hidden elements. Uncertainty estimation of XCT is still in development, and studies typically use high-resolution calibrated devices such as focal variation microscopes (FVMs) as a reference, focusing on certain parts of the lattice but not the whole structure. In this paper, an estimation of the accuracy of XCT evaluation of a complete lattice structure in comparison to a higher-resolution reference device (FVM) is presented. Experimental measurements are taken on ad hoc designed test objects manufactured in polyamide 12 (PA12) using selective laser sintering (SLS), optimized for the evaluation on both instruments using different cubic-based lattice typologies. The results confirm higher precision on XCT evaluation in both qualitative and quantitative analysis. Even with a lower resolution, XCT is able to characterize details of the surface such as re-entrant features; as well, standard deviations and uncertainties in strut diameter evaluation remain more stable in all cells in XCT, identifying on the other hand reconstruction problems on FVM measurements. Moreover, it is shown that, using XCT, no additional evaluation errors were found in inner cells, suggesting that the measurement of external elements could be representative of the whole structure for metrological purposes.

## 1. Introduction

Additive manufacturing (AM) is an innovative and relatively recent industrial technology in which material is added layer-by-layer to create parts. Its main characteristic is versatility: due to its manufacturing principle, in contrast to traditional technologies, complex geometries such as freeform surfaces, hidden cavities, or lattice structures can be produced [1,2,3,4]. The development of AM technologies has increased the quality of the parts produced, from only formal prototypes to final products with optimal mechanical and thermal properties, whether the material used is polymer, metal, ceramic, or even reinforced/compound materials [5,6,7]. AM concept groups have different typologies: fused deposition modeling (FDM), selective laser sintering (SLS), multijet, etc., each one with singular characteristics.

In the industry, the introduction of lattice structures in newly developed products is particularly interesting [8]. These features are designed to replace solid parts, maintaining the same mechanical properties of the object and reducing the amount of material used and the total weight; in conclusion, they can create more cost-effective designs. However, as with most complex geometries produced by AM, quality control in terms of dimensional accuracy becomes difficult as lattices are not easily accessible using common metrological devices (such as tactile machines and optical microscopes).

Here, X-ray computed tomography (XCT) has become a solution for the inspection of complex geometries and hidden features [9,10]. XCT is based on the reconstruction of a 3D volume through 2D X-ray images acquired along a 360° rotation of the measurand. Volumes obtained by XCT not only includes the information on the surface of the object but also the inner parts; thus, it is possible to inspect and characterize non-accessible areas and inner cavities. Its application in industrial metrology [11,12,13] has required the improvement of the acquisition techniques in order to obtain high-precision volumes optimal for the measurement of macro geometries and micro features such as the surface roughness [14,15,16,17].

As a newly metrological technique, XCT still has disadvantages to overcome. Due to the high number of factors affecting the accuracy of measurements, uncertainty calculations are challenging, and the normative that regulates them is still in development. Currently, the substitution method [18,19] is the main procedure used to calculate uncertainties in XCT evaluation; however, it requires the usage of a reference device to calibrate the test objects and make the correspondent comparisons [20,21,22]. For certain cases, as indicated before, those reference measurements are not possible due to the geometry and the range of evaluation of the device.

### 1.1. State of the Art

One of the most commonly used devices for the obtention of high-quality surfaces is the focal variation microscope (FVM), which allows obtaining 2.5D measurements with resolutions up to nanometers. Its application to surface roughness evaluation and texture characterization in AM parts is widespread [23,24], and it has been used as a reference instrument for the comparison of XCT measurements [15,16]. The main disadvantage of this technique is the limitation in the range of evaluation, which is reduced to a few millimeters; reconstructing 3D surfaces is highly time-consuming, as several local measurements are required to be used and oversampled.

XCT, as stated before, has been a huge innovation due to its versatility in measuring macro and micro geometries and the inspection of complex geometries or internal features [25,26,27,28]. Although post-processing of the information is needed, the amount of metrological data obtained is superior to other instruments in less time. It is commonly used for the metrological evaluation of multi-material parts [29,30] and composites [31,32,33]. However, as stated before, the traceability of XCT measurements is still in development, as well as the normative, which defines the procedures for the uncertainty calculations. This is the reason for the common usage of reference devices with better traceability for the verification of the results [34,35,36].

Several studies have been performed in the field of lattice characterization using XCT. In [22], a reference object was designed to estimate uncertainties in lattices through a variation of the substitution method. The concept of the theoretical supplemental surface was used in [37] for the verification and tolerancing of an AM lattice. Also, the resolution of XCT measurements has been studied [38] as a factor in XCT evaluation accuracy.

Lattice evaluation is challenging for other methods different from XCT, as stated before; however, in some cases, a comparison has been performed by using reference devices as coordinate measuring machines (CMMs) [39] for the measurement of outer faces of the lattice (as it was not possible to reach the inside) or FVM [40], with the objective of replicating the surface texture of the outer lattice struts.

Single-strut measurements are also used [41] for a general characterization of the complete part, in which the orientation of the strut is evaluated as a parameter. Zanini et al. [42] explored the application of two different methods for uncertainty calculation in lattice structures, with both using an additional calibrated reference standard as a test object along with a lattice part.

To sum up, efforts have been focused on the study of certain factors of XCT measurement (orientation, resolution, surface roughness, outer faces) or the traceability of XCT evaluations through a reference-calibrated object, which typically differs from a real lattice.

### 1.2. Research Aim

In this paper, the main aim is to evaluate the precision of XCT when measuring a realistic lattice structure through an intercomparison with a surface characterization reference device. FVM, the instrument used for this study, is able to obtain high-quality 3D surfaces but has the limitation of the range of evaluation; here, an experiment is made using a test object adapted for the measurement by both FVM and XCT, simulating a 4 × 4 × 4 cubic lattice.

Polymeric additive manufacturing has been selected for the manufacturing of the parts. Typically, studies are based on metal lattice structures; however, polymeric lattice structures also have a great interest in industry as they are more cost-effectively produced. Additionally, the evaluation of polymers by XCT requires different machine settings, and therefore, the results of measurements performed on metal parts cannot be extrapolated. Selective laser sintering (SLS) is the AM process selected for the manufacturing of the parts, choosing polymer polyamide 12 (PA12) for all the test objects. SLS is an appropriate AM process for the production of complex geometries as there is no need for support structures, as the powder itself acts as a support layer-by-layer. Other processes, as Polyjet, have been considered, as they also do not require support structures and provide good part accuracy. Although parts manufactured by SLS are more likely to be more porous, the cleaning post-processing of small and complex features is easier on SLS, so the danger of failure of the lattices is reduced.

Three typologies of parametric lattices were selected for the experiment, all cubic-based, and a dimensional measurement and comparison of the struts were performed along with a qualitative surface evaluation.

## 2. Materials and Methods

In this section, a description of the test objects designed to carry out the experiments and the methodology followed to support their evaluation is presented.

### 2.1. Test Objects

Test objects were designed for the optimization of the measurements both in XCT and FVM. Three lattice configurations were used for the design of the probes, according to the most common typologies and the most suitable for the experiment: body-centered (BCC), face-centered (FCC), and body-centered with additional vertical struts (BCCZ). Each individual probe included 4 cubic cells of 5 mm length and 1 mm diameter struts. Criteria for the selection of cell size and strut diameter were used to obtain a proper diameter/length ratio in the struts: here, 1:5 ratio was considered appropriate. With this ratio, amount of material is significantly reduced in comparison to a solid cube, and the post-processing of the parts is facilitated. Higher ratio would not be realistic for a lattice structure, as mechanical characteristics of the part would have been considerably affected.

**Figure 1:**
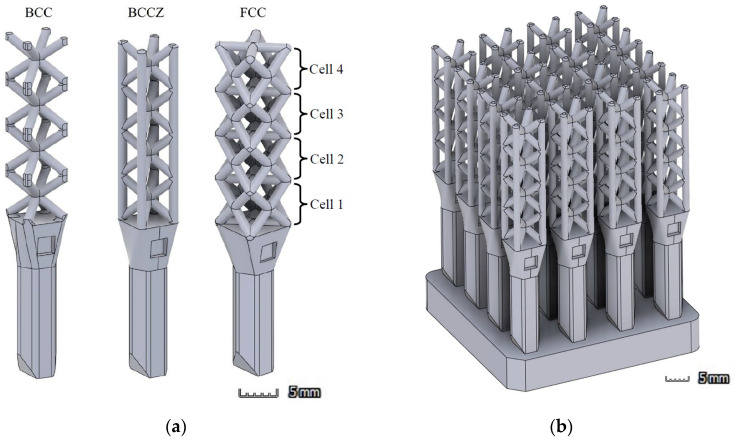
(**a**) Individual probes of each cell typology: body-centered cubic (BCC), body-centered cubic with vertical struts (BCCZ), and face-centered cubic (FCC); (**b**) 4 × 4 × 4 assembly (BCCZ).

An assembly was designed for each typology, which included several individual probes, connected by a solid base (Figure 1). The intention of this assembly is to simulate a 4 × 4 × 4 lattice structure for the measurements in XCT; however, in this configuration, it was not possible to reach the inner cells using Alicona (Graz, Austria), so the solid base was removed after the XCT evaluations to measure each probe individually in the microscope.

Manufacturing of the test objects was carried out using SLS device (Lisa Pro, Sinterit, Kraków, Poland) in polymer polyamide 12 (PA12). Post-processing was applied after the manufacturing by cleaning the parts with compressed air and abrasive dust, as recommended by Sinterit, to remove unfused particles in the surface.

### 2.2. Methodology

**Figure 2:**
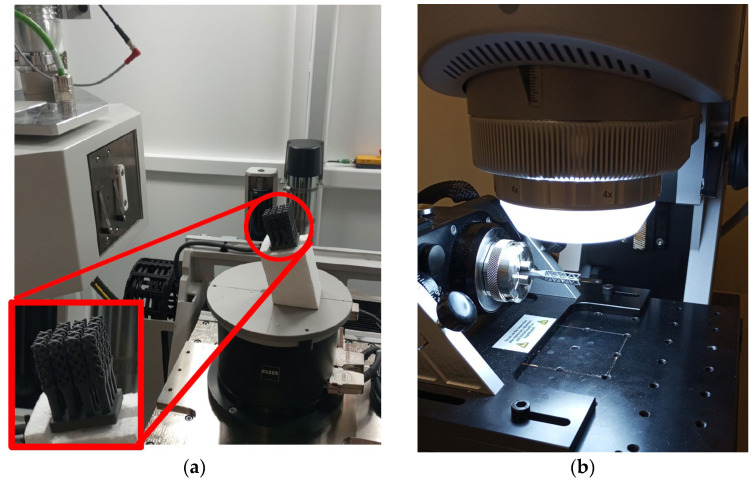
(**a**) FCC assembly (circled in red, detailed in lower left corner) mounted in the XCT platform; (**b**) BCCZ individual probe placed in the accessory rotary plate of FVM.

First evaluation of the assemblies was performed by XCT Zeiss Metrotom 800 G3/225 kV, using integrated software Metrotom OS 3.12 (Zeiss, Oberkochen, Germany). A total of 5 iterations were taken for each assembly. After XCT evaluation, individual probes were separated from the solid base and measured by focal variation microscope—InfiniteFocusSL, from Alicona—using an incorporated rotary plate that allows obtaining a 360° scan along a horizontal axis and integrated software IfMeasure Suite 5.3.6 (Alicona Imaging, Graz, Austria). Disposition of the test objects for the evaluation in XCT and FVM is displayed in Figure 2.

Settings used for the evaluation in XCT and FVM are displayed in Table 1. Same settings were used for all parts.

Resolution and time elapsed are the two parameters resulting from the adjustment of the process settings of each method. For FVM, sampling resolution is inversely proportional to time elapsed, while in XCT, the resolution obtained is the minimum achievable for the complete characterization of the test object. This depends on the geometrical magnification, a property resultant from the source-to-object distance (SOD) and source-to-detector distance (SDD). Magnification in this case could have been improved by local tomographies; however, time elapsed would have increased exponentially with no significant accuracy gains.

STL files were extracted for each probe for dimensional evaluation and intercomparison XCT-FVM using software Zeiss Inspect X-ray Pro 2023.3.0 and VG Studio Max 3.4.2. In XCT evaluation, surface determination of the parts was performed in software VG Studio Max 3.4.2, in Advanced mode, selecting a local gradient threshold with a search distance of 4 voxels, with no extra post-processing. Geometry and material of the test objects do not imply a challenge in terms of XCT process settings adjustments or surface determination; therefore, the aim was to automate as much as possible the surface-determination process in order not to have any influence on the dimensional results.

## 3. Results

In this section, details of the results obtained in the evaluation of the test objects are shown, including a first qualitative comparison of the STL obtained (Section 3.1) and various analyses of the dimensional measurements of the lattice struts (Section 3.2).

### 3.1. STL Quality Comparison

The first qualitative analysis of the lattice surfaces obtained by FVM and XCT was performed. STL files were extracted from each FVM and XCT measurement and imported using the software VG Studio Max 3.4.2 for the evaluation.

**Figure 3:**
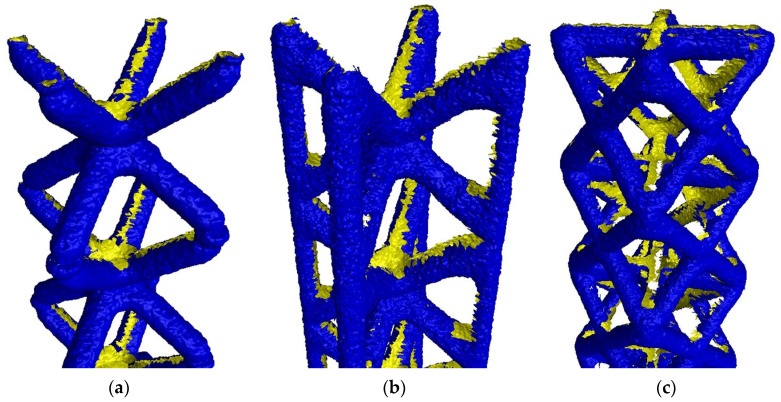
STL files obtained for each lattice typology in FVM measurements. Outer surface (blue) and holes (yellow) are displayed. (**a**) BCC; (**b**) BCCZ; (**c**) FCC.

In Figure 3, STL files of a single probe from each lattice typology in FVM measurements are shown.

The first visual observation indicates that the reconstructed 3D files are not complete in all cases; however, BCC probes show considerably fewer holes. These errors are located mainly in internal zones, which are not possible to characterize by FVM as remaining “hidden” by outer elements. Areas with no data have registered artifacts and deformations in the edges.

**Figure 4:**
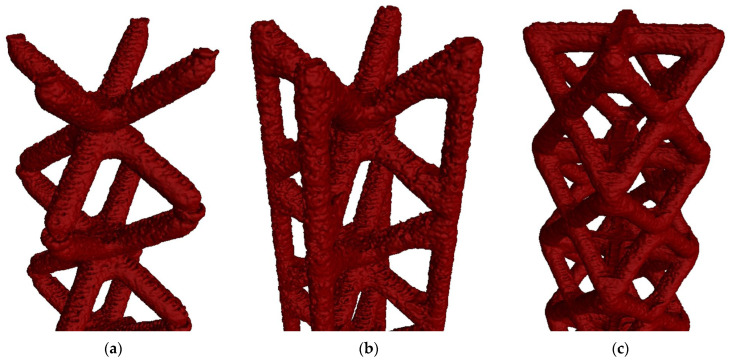
STL files obtained for each lattice typology in XCT measurements. (**a**) BCC; (**b**) BCCZ; (**c**) FCC.

In Figure 4, STL files of the XCT reconstruction of single probes for each typology are displayed.

**Figure 5:**
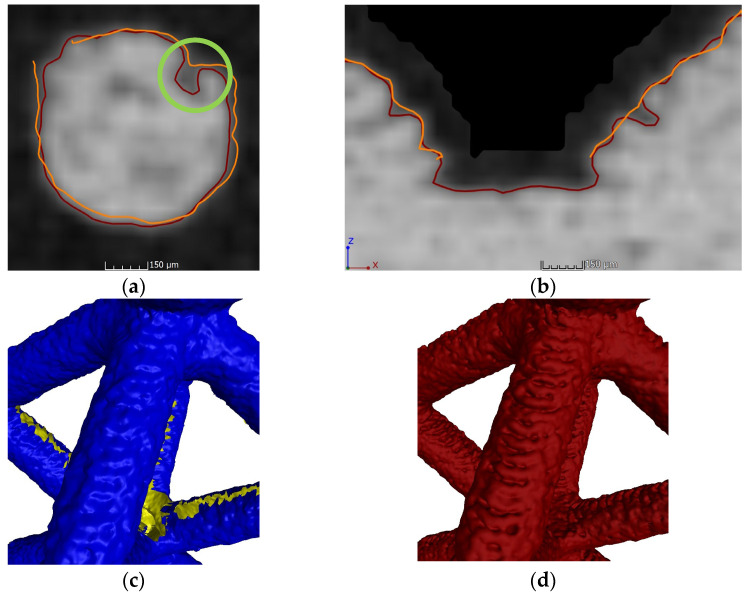
A 2D slice comparison of FVM (orange) and XCT (red) surface reconstruction over grayscale map. (**a**) BCCZ vertical strut; (**b**) FCC transversal cut of a node; (**c**) 3D details of the FVM surface of a BCC strut (yellow areas represent holes); (**d**) 3D details of the XCT surface of a BCC strut.

The XCT evaluation allowed us, as expected, to obtain complete STL files without holes in the inner areas; as stated before, this technique allows us to inspect hidden elements and inner features. Therefore, it has been able to reach zones that FVM is not capable of characterizing. However, this first look does not allow us to fully understand the level of detail of both devices. A close-up evaluation through 2D sections was performed. In Figure 5, examples of 2D slices of both XCT and FVM surfaces with a grayscale XCT background are shown, along with 3D details of the surfaces.

In these 2D slices and 3D details, it is possible to see that the XCT surface has more microscopical details at a surface roughness level and at some features that, even though they are not internal elements, are not directly on sight; these elements are called re-entrant features (circled in green in Figure 5a) and are only possible to be measured by XCT [43]. Holes in the surface and the edge defects are evident, as seen in Figure 4.

The first visual analysis suggests that the quality of the 3D data obtained by XCT is higher than by FMV. Inner areas are not adequately characterized by FVM, showing errors in the edges of the holes that could affect the measurements of the struts. Also, some microscopic details and re-entrant features are only possible to be obtained by XCT; although having better resolution, it is possible that problems have occurred in FVM results due to the 360° reconstruction. Here, it has been shown that the cell type was relevant in FVM evaluation, as cell types with a higher amount of surface area that is not in direct sight have bigger reconstruction errors. In XCT, as this technology is able to characterize internal and hidden elements, this problem is not observed. To confirm the suggestions obtained in this analysis, a quantitative evaluation was performed through dimensional measurements of the lattice struts.

### 3.2. Dimensional Measurements

The results were obtained from the dimensional measurements of the individual struts of the lattices. The mean values of each cell were intercompared for each lattice typology (Section 3.2.1) and according to the cell position (Section 3.2.2). For this purpose, the software Zeiss Inspect X-ray Pro 2023.3.0 was used.

#### 3.2.1. Single Probe

**Figure 6:**
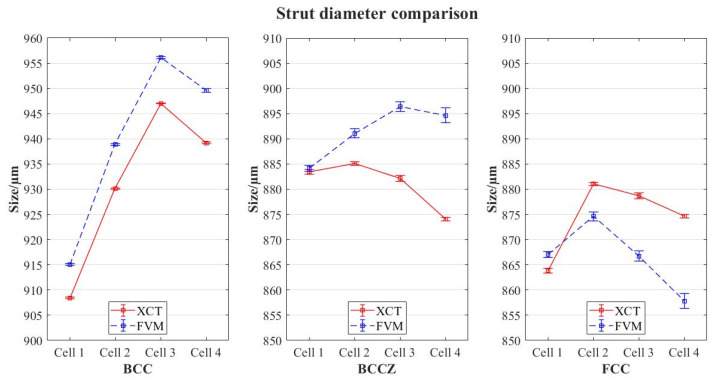
Strut diameter comparison for each lattice typology with standard deviations.

The first analysis was performed by comparing the evaluation of a single probe of each lattice typology using both devices. The mean values of the struts’ diameters were grouped by cell unit, following the scheme shown in Figure 1a. A comparison of the mean values obtained from the XCT and FVM evaluation for each lattice typology are displayed in Figure 6, including standard deviations of the results (error bars).

The results show an increase in the deviation of the values between both devices for cells located in higher positions; this becomes more evident for BCCZ and FCC, but the affection is different (diameters in FVM evaluation for FCC are smaller, while those for BCCZ are bigger). The standard deviation (σ) of the mean results is summarized in Table 2.

Additionally, expanded uncertainty calculations were made according to the procedures indicated in the specific normative for each device:

- ISO 15530-3:2011 [18] for FVM measurements. ISO 15530-3:2011 defines general calculations for the uncertainty estimation of a metrological device. The equation used for the calculation of the expanded uncertainty *U* is the following:
  (1) $$U_{FVM}=k*\sqrt{u_{cal}^{2}+u_{p}^{2}+u_{w}^{2}+u_{b}^{2}},$$
  where *k* is the coverage factor (*k* = 2 for a 95% confidence); *u_cal_* is the standard uncertainty of measurement due to the uncertainty of calibration of the calibrated workpiece, as stated in the calibration certificate; *u_p_* is the standard uncertainty of measurement due to the measurement process, (standard deviation (σ) of the repeated measurements); *u_w_* is the standard uncertainty of measurement due to variations in materials and production (due to variations in, e.g., the coefficient of expansion, form errors, roughness, elasticity, and plasticity); and *u_b_* is standard uncertainty of the measurement of the correction of the systematic error b between the values, *yi*, indicated by Alicona and the calibrated value, *x_cal_*, of the calibrated workpiece.
- VDI/VDE 2630-2.1 [19] for XCT measurements. As stated before, there is still no standard for the determination of the uncertainties in XCT evaluation; however, this directive has good explanations of the factors to consider. Also, recommendations suggested in [44] are followed.

VDI/VDE 2630-2.1 recommends an addition of parameter *u_drift_*, which is the standard uncertainty of measurement due to the change (drift) in workpiece shape due to the calibration referred to. Here, as the measurements performed by both devices are performed one after the other with no time lapse, and no changes in the workpiece are supposed, *u_drift_* could be neglected. It is also worth mentioning that, for XCT uncertainty calculations, a maximum permissible error (MPE) = 4 µm, considering a rectangular distribution, was used as it was provided by the manufacturer of the XCT device. The equation, thus, will remain as following for XCT measurements:

(2) $$U_{XCT}=k*\sqrt{u_{cal}^{2}+u_{p}^{2}+u_{w}^{2}+u_{b}^{2}+u_{drift}^{2}},$$

**Figure 7:**
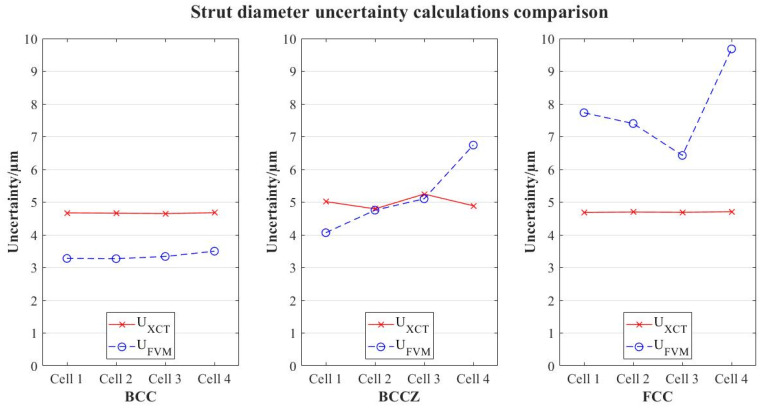
Comparison of uncertainties in strut diameters for each lattice typology.

The results of the uncertainty calculations registered for the strut diameter measurements are shown in Figure 7.

The standard deviations registered are considerably higher for FVM measurements; also, it is shown that, even with considerably lower resolution, uncertainty of the results is similar or even lower for XCT measurements. This, combined with trends observed in Figure 4, the results suggest that (i) repeatability of XCT results is higher and (ii) reconstruction problems occur in FVM characterization. FVM measurements are taken along a horizontal axis in which the single probe is aligned. As the alignment is not perfect, rotation of the probe is not totally symmetric; therefore, the further the cell is from the clamping of the probe, the higher the asymmetry, creating a “cone effect”. The numerical results confirm that this affects the reconstruction of the volume for FVM, as deviations are higher in the upper cells. In XCT, however, this effect does not happen, as the standard deviations and the uncertainties are similar regardless of the position of the cell in the probe.

This reconstruction problem in FVM affects differently depending on the geometry of the lattice. BCC cells show smaller deviations, although the trend stated before (higher position, higher errors) is still followed; this suggests that the more surface reachable by the microscope, the higher the correlation with XCT results.

#### 3.2.2. Cell Position—4 × 4 × 4 Structure

**Figure 8:**
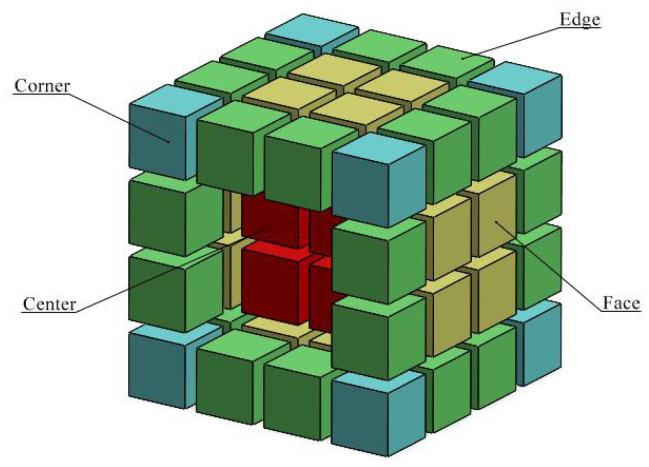
Cell position groups divided by colors.

A second analysis was performed regarding strut diameter evaluation. In a complete lattice, cells were divided according to their location in the 4 × 4 × 4 structure, as it is shown in Figure 8, and grouped in 4 different positions: corners, faces, edges, and centers.

**Figure 9:**
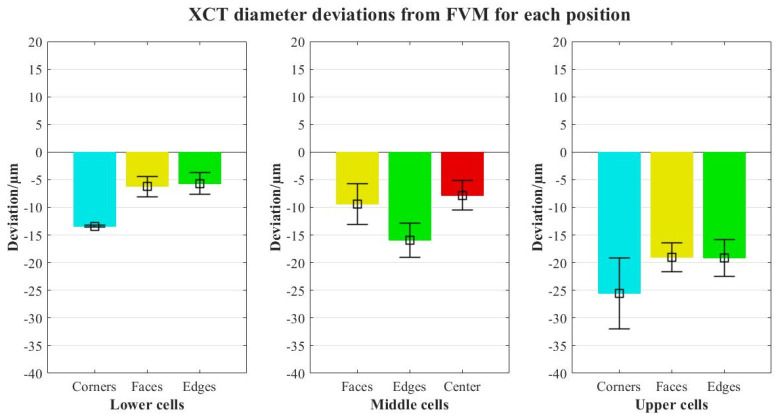
Comparison of the XCT deviations of the struts’ diameters from FVM for each cell location along the structure.

The objective was to check for different trends in the deviations XCT-FVM in contrast to the results of the single-probe evaluation. The BCCZ lattice was chosen for this analysis, as the lattice density is higher, and it can create higher noise levels in the measurement of inner cells. The results are shown in Figure 9.

The results are divided into lower, middle, and upper cells considering single-probe analysis segmentation. The values obtained show similar trends as previously, with higher deviations in upper cells. Regarding positions, outer cells have higher errors (also worse repeatability in terms of the standard deviation of results) than inner cells; this is an important point, as it suggests that no additional measurement errors are found in inner cells, and therefore in the case that reference measurements are required for any metrological analysis (accuracy, uncertainties, etc.), outer cell characterization is representative enough for the whole structure.

## 4. Discussion

The analysis of the measurements taken shows, as expected, better results for XCT in all aspects. Although a 3D reconstruction of the single probes by the microscope is possible with acceptable accuracy, the better repeatability of the XCT surfaces obtained is demonstrated by smaller standard deviations and better uncertainties in strut diameter evaluation and higher-quality STL files. Although having better resolution, FVM is not capable of characterizing certain details, such as re-entrant features, due to its limiting optical measuring principle; in XCT, however, these non-visible elements are reachable, therefore obtaining surfaces closer to the real part. Of course, the aim of this experiment is not to evaluate the precision of FVM in lattice structure evaluation as it is not the most suitable device for this purpose. Still, it is worth pointing out that compared to the reference instrument, which had a remarkably better resolution (up to hundreds of nanometers) when acquiring surface data, XCT obtained more repeatable results, which leads to the suggestion of better precision. In addition, it is confirmed that cell type influences the accuracy of the FVM evaluation method as the area proportion which is directly visible by the microscope is not equal for all typologies; however, in XCT, there is no such problem, as this technique is able to characterize hidden surfaces.

Another aspect to consider is the non-variability of the XCT results no matter the position of the cell in the 4 × 4 × 4 structure, apart from the deviations found caused by the reconstruction errors in FVM. As stated in previous studies [42], it is challenging to compare inner cell results with reference devices because XCT is the only non-destructive method able to characterize hidden parts. Thus, it is relevant that the XCT accuracy does not decrease for inner cells; this suggests that a reference measurement of external lattice elements by a reference device is enough for a performance evaluation of a complete XCT lattice inspection. This must be taken with caution, as this experiment is performed in a single material (polymer) and with a particular methodology; for other materials or reference devices, further experiments should be required.

## 5. Conclusions and Future Work

In this paper, an approach to the definition of true precision in X-ray computed tomography (XCT) characterization of polymeric lattice structures through a metrological evaluation is presented. An experimental framework is settled, using a focal variation microscope (FVM) as a calibrated reference device for the intercomparison with the XCT measurements; this instrument was selected due to its higher resolution and its common usage in surface 3D data acquisition. Ad hoc test objects were designed for the optimization of measurements by both devices, simulating 4 × 4 × 4 structures composed of cubic-based lattice cells organized in three typical configurations: body-centered cubic (BCC), body-centered cubic with vertical struts (BCCZ), and face-centered cubic (FCC). A first qualitative analysis of the surface obtained from the probes was performed, along with a metrological evaluation of the diameters of the struts considering single-probe evaluation (Section 3.2.1) and the complete structure for cell position error analysis (Section 3.2.2).

The results show higher repeatability of the values obtained in XCT measurements of the diameter of the struts, with significantly lower standard deviations and good uncertainty values. A trend was observed in FVM evaluation, as higher deviations from XCT as well as lower repeatability were registered for upper cells. Errors are most likely caused by reconstruction of the volume, as the rotation axis is not totally aligned with the horizontal axis; this “cone-effect” is less evident in the BCC lattice, matching with the higher amount of data obtained in the characterization.

Analysis of the deviations according to cell position along the 4 × 4 × 4 structure has revealed similar trends, with no additional differences apart from reconstruction errors in FVM mentioned before. This shows that no higher dimensional errors are found in the XCT evaluation of inner cells of the lattice, therefore suggesting that the accuracy of inner cell XCT measurement does not decrease, and reference measurements taken in outer cells may be representative of all of the structure. However, this must be taken with caution, as this experiment was performed with a particular methodology and for polymers.

As future work, further research should be performed for other materials (such as metals) to complement this knowledge and to find out if these conclusions obtained could be generalized for all cases. Additionally, studies could be expanded to other lattice types, such as gyroids, diamond shapes, or organic forms, to verify the methodology.

## Figures and Tables

**Table 1:** Settings and parameters of the evaluation in each device.

	XCT	FVM
Voltage/kV	70	-
Current/µA	478	-
Physical filter	No	-
Projections	1700	-
Exposure time/ms	667	27.5
Contrast ^1^	-	0.45
Magnification	4.0	4× AX (Lens)
Resolution/µm	34.26 (Voxel size)	0.13 (Vertical), 8.5 (Lateral)
Time elapsed/min	14–16	70–75 (each probe)

^1^ non-dimensional contrast coefficient provided by software IfMeasure.

**Table 2:** Standard deviation (σ) of mean results of strut diameter evaluation for each case.

	Cell 1		Cell 2		Cell 3		Cell 4	
	XCT	FVM	XCT	FVM	XCT	FVM	XCT	FVM
*σ_BCC_*	0.32	0.37	0.28	0.35	0.23	0.49	0.33	0.72
*σ_BCCZ_*	0.97	1.26	0.62	1.76	1.23	1.99	0.79	2.97
*σ_FCC_*	0.36	3.52	0.39	3.34	0.37	2.79	0.42	4.57

## Data Availability

Data are contained within the article.

## Abbreviations

The following abbreviations are used in this manuscript:

XCT	X-ray computed tomography.
FVM	Focal variation microscope.
PA12	Polyamide 12.
SLS	Selective laser sintering.
CMM	Coordinate measuring machine.
BCC	Body-centered cubic.
BCCZ	Body-centered cubic with vertical struts.
FCC	Face-centered cubic.
σ	Standard deviation.
MPE	Maximum permissible error.
